# Influence of *DNMT3A* R882 mutations on AML prognosis determined by the allele ratio in Chinese patients

**DOI:** 10.1186/s12967-019-1959-3

**Published:** 2019-07-10

**Authors:** Xiao-Qing Yuan, Peng Chen, Yin-Xiao Du, Ke-Wei Zhu, Dao-Yu Zhang, Han Yan, Han Liu, Yan-Ling Liu, Shan Cao, Gan Zhou, Hui Zeng, Shu-Ping Chen, Xie-Lan Zhao, Jing Yang, Wen-Jing Zeng, Xiao-Ping Chen

**Affiliations:** 1 Department of Clinical Pharmacology, Xiangya Hospital, Central South University, Changsha, 410008 Hunan People’s Republic of China; 2 Institute of Clinical Pharmacology, Hunan Key Laboratory of Pharmacogenetics, Central South University, Changsha, 410078 Hunan People’s Republic of China; 3 Department of Hematology, Xiangya Hospital, Central South University, Changsha, 410008 Hunan People’s Republic of China; 4 Department of Pharmacy, The First Affiliated Hospital of Zhengzhou University, Zhengzhou, 450052 Henan People’s Republic of China; 5 National Clinical Research Center for Geriatric Disorders, Xiangya Hospital, Central South University, Changsha, 410008 Hunan People’s Republic of China

**Keywords:** Acute myeloid leukemia, *DNMT3A*, R882 mutations, Allele ratio, Prognosis, Aclarubicin

## Abstract

**Background:**

The influence of *DNMT3A* R882 mutations on adult acute myeloid leukemia (AML) prognosis is still controversial presently. The influence of R882 allele ratio on drug response and prognosis of AML is unknown yet. Besides, it is obscure whether anthracyclines are involved in chemoresistance resulted from R882 mutations.

**Methods:**

*DNMT3A* R882 mutations in 870 adult AML patients receiving standard induction therapy were detected by pyrosequencing. Associations of the mutants with responses to induction therapy and disease prognosis were analyzed.

**Results:**

*DNMT3A* R882 mutations were detected in 74 (8.51%) patients and allele ratio of the mutations ranged from 6 to 50% in the cohort. After the first and second courses of induction therapy including aclarubicin, complete remission rates were significantly lower in carriers of the *DNMT3A* R882 mutants as compared with R882 wildtype patients (*P* = 0.022 and *P* = 0.038, respectively). Compared with R882 wild-type patients, those with the R882 mutations showed significantly shorter overall survival (OS) and disease-free survival (DFS) (*P* = 1.92 × 10^−4^ and *P* = 0.004, respectively). Patients with higher allele ratio of R882 mutations showed a significantly shorter OS as compared with the lower allele ratio group (*P* = 0.035).

**Conclusion:**

Our results indicate that the impact of *DNMT3A* R882 mutations on AML prognosis was determined by the mutant-allele ratio and higher allele ratio could predict a worse prognosis, which might improve AML risk stratification. In addition, *DNMT3A* R882 mutations were associated with an inferior response to induction therapy with aclarubicin in Chinese AML patients.

**Electronic supplementary material:**

The online version of this article (10.1186/s12967-019-1959-3) contains supplementary material, which is available to authorized users.

## Background

Acute myeloid leukemia (AML) is a clonal but heterogeneous malignancy characterized by deregulated proliferation and inhibited differentiation of hematopoietic progenitors, as well as highly diverse pathogenesis, response to chemotherapy and clinical prognosis [1]. Cytogenetic or chromosomal aberrations, such as *AML1*-*ETO* and *CBFβ*-*MYH11* fusion genes, are observed to play a role in AML pathogenesis and have prognostic significance [2]. The presence of somatic mutations in genes including *NPM1* (Nucleophosmin 1), *CEBPA* (CCAAT/enhancer binding protein alpha), *c*-*KIT* (tyrosine-protein kinase Kit) and *FLT3* (Fms-like tyrosine kinase 3) can also promote myeloid leukemogenesis and influence the prognosis of AML [3]. Combined chemotherapy with one of the anthracyclines (daunorubicin, idarubicin, aclarubicin or mitoxantrone) for 3 days and cytarabine for 7 days, known as “7 + 3” regimen, remains the standard induction treatment for AML except for the French–American–British (FAB) M3 subtype [4]. The complete remission (CR) rate is about 70–80% for patients under 60 years and 40–50% for patients over 60 years after induction therapy [5]. The 5-year survival rate for adult patients with AML is low, especially for those aged 65 or older [6, 7]. Disease heterogeneity may partly account for the interindividual difference in drug response and disease prognosis.

Epigenetic modification also plays important roles in normal hematopoiesis through regulation of cellular processes, and the loss-of-function of epigenetic modifiers may contribute to the etiology and development of AML. The *DNMT3A* (DNA methyltransferase 3 alpha) gene encodes a DNA de novo methyltransferase DNMT3A that regulates gene expression through methylation of the cytosine residue of CpG dinucleotides. The gene has recently garnered attention because of its frequent mutations in a variety of adult hematologic malignancies, often occurring as early events during leukemogenesis [8]. *DNMT3A* mutations could be detected in approximately 20% of AML cases and 34% of cytogenetically normal AML cases, and about 65% of the mutations were alterations from arginine 882 to histidine (R882H) or cysteine (R882C) within the catalytic domain of the protein [9, 10].

The R882H mutation could reduce approximately 80% methyltransferase activity in a dominant negative manner, but might not directly affect the property of cytosine methyltransferase [11–13]. The mutant protein profoundly inhibits the wildtype protein through disrupting its ability to form tetramers, which is a substantially more active form of the enzyme [13]. Challen et al. reported that loss of *DNMT3A* either increased or decreased DNA methylation levels at distinct loci, most of which are involved in hematological malignancy [14]. The association between hypermethylation of promotor CpG islands and mutated *DNMT3A* was observed in AML [15]. Genome-wide hypomethylation in patients with the R882 mutations is also observed, especially for the genes encoding the HOX family proteins [13, 15, 16]. Recently, we and others reported that the *DNMT3A* mutations are associated with adverse survival outcomes and poor prognosis for AML patients [10, 17–20]. Nevertheless, more evidence is claimed to confirm the clinical relevance of *DNMT3A* mutations in AML for clinical decision [21, 22].

Results from clinical studies showed that AML patients with *DNMT3A* mutations had inferior outcomes after standard-dose daunorubicin treatment [19, 22–24]. In addition, dose-escalated daunorubicin therapy could overcome the negative impact of *DNMT3A* mutations [25–27]. The previous reports indicated that *DNMT3A* R882 mutations might enhance chemoresistance to induction regimens including anthracyclines. Kim et al. found that AML patients with high *FLT3*-*ITD* (*FLT3* internal tandem duplication) allelic ratio or long ITD length had a significantly worse prognosis [28]. An impact of *RUNX1* (runt related transcription factor 1) allele dosage on gene expression profile and glucocorticoid sensitivity was also observed in AML [29]. A recent study also showed associations of high *NPM1* variant allele with shortened OS and EFS in AML [30]. The series of studies suggested that the allelic ratio of somatic mutations in AML could affect the biological properties of tumor and might be one of the critical factors influencing disease prognosis.

In spite of the association between *DNMT3A* R882 mutations and worse outcome, it is obscure whether the mutations are associated with response to anti-leukemic therapies, and whether *DNMT3A* mutant types at amino acid 882 or allele burden influence prognosis of AML. Therefore, we investigated the relevance of *DNMT3A* R882 mutation types and allelic ratio to chemotherapy efficacy and prognosis, and performed subgroup analysis according to induction regimens in 870 Chinese AML patients.

## Methods

### Study design and patient population

In this cohort study, 870 patients with non-M3 AML were enrolled at Xiangya Hospital, Central South University between May 2009 and July 2018. Patients aged 14 years or older, diagnosed with AML according to the WHO criteria, and received cytarabine combined with anthracyclines for “7 + 3” induction chemotherapy were enrolled. Exclusion criteria included acute promyelocytic leukemia (FAB-M3 AML), therapy-related AML (T-AML), acute mixed lineage leukemia, or accompanied by other cancer or serious diseases. Treatment options for AML are described previously [31]. Demographic and clinical information of the patients were collected from medical records and regular outpatient review. Patients were regularly questioned about clinical events once every 3 months by telephone, which ended on Sept. 30th, 2018. The study was conducted in accordance with the Declaration of Helsinki and approved by the Ethics Committee of Institute of Clinical Pharmacology of Central South University (No. CTXY-120025-2) and the Chinese Clinical Trial Register (ChiCTR-PPC-14005297). Written informed consents including genetic information sharing with investigators were obtained from each participant before enrollment.

### Clinical end points and response criteria

The primary endpoints were drug response, overall survival (OS) and disease-free survival (DFS). The criterion of CR was defined as follows: less than 5% blasts and no blasts with Auer rods in bone marrow; absence of extramedullary disease; absolute neutrophil count > 1 × 10^9^/L and platelets ≥ 100 × 10^9^/L independent of transfusions [32]. Treatment-related mortality (TRM) was defined as death within 28 days after initiation of induction therapy because early death in AML patients frequently occurred during 4 weeks after induction therapy [33]. Those failed to obtain CR and patients suffered from TRM after induction chemotherapy were categorized as the non-CR group. Disease relapse was defined as the presence of more than 5% of blasts in the bone marrow or the reappearance of blast cells in peripheral blood or the development of extramedullary disease. OS was the length of time from AML diagnosis until death caused by any reason. For patients achieving CR, DFS was calculated from date of the first remission until the date of relapse or death. Patients who underwent hematopoietic stem cell transplantation (HSCT) after achievement of CR were censored at the date of HSCT for both OS and EFS. For patients with no disease relapse or death events at the end of the follow-up, the last follow-up date was regarded as censored data for survival.

### Detection of somatic mutations in AML

Peripheral venous blood or bone marrow samples were collected from newly diagnosed AML patients. Genomic DNA was extracted using E.Z.N.A.^®^ SQ Blood DNA Kit II (Omega Bio-Tek company, USA) according to the manufacturer’s instructions and stored at − 80 °C until use. *FLT3*-*ITD* mutations were detected as described elsewhere [34]. Briefly, DNA fragment between the 14th and the 15th exons of *FLT3* gene was amplified by polymerase chain reaction (PCR), and the PCR products were then electrophoresed through 2% agarose gels. The PCR product of 328 bp was from the *FLT3* wildtype allele. *NPM1* and *DNMT3A* R882 mutations were detected using pyrosequencing, and R882 mutant allele ratio was also calculated. In detail, DNA segments containing the 12th exon of *NPM1* or the 23th exon of *DNMT3A* were amplified through PCR in a final reaction volume of 50 µL, which contained 41 μL sterile double-distilled water, 5 μL PCR buffer, 2 μL DNA, 1.5 μL dNTP, 0.5 μL DNA polymerase, and 0.05 nM of each primer. Thermal cycling procedure for PCR was as follows: degeneration at 95 °C for 5 min; 35 cycles of 95 °C for 30 s, 57 °C for 35 s and 72 °C for 30 s; a final extension at 72 °C for 7 min. After verification by agarose electrophoresis, the amplified fragments were analyzed by pyrosequencing on the PyroMark Q24 Advanced platform (Qiagen, Germany) with the pyrosequencing primers. Sequences of the primers were shown in Additional file 1: Table S1.

### Statistical analysis

Statistical analyses were performed with the software SPSS 18.0. Pearson Chi Square test, Continuity correction or Fisher’s exact test were applied to compare differences in chemosensitivity to one or two cycles of induction therapy, toxicity, and other categorical data between *DNMT3A* R882 genotype groups. Odds ratios (OR) were used as indicators to evaluate relative risk level of non-CR. Continuous variables between *DNMT3A* R882 genotype groups were compared using independent Student’s T test or Mann–Whitney U test. Patients with *DNMT3A* R882 mutations were divided into high and low mutation allele ratio groups by using median of allelic ratio as the cut-off value. Logistic regression analysis was performed to estimate the relative risk of non-CR adjusted for AML prognostic factors including age, WBC count, and risk stratification. Survival data was assessed by Kaplan–Meier method and difference between groups were compared by the log-rank test. Hazard ratios (HR) for OS and EFS were estimated by Cox proportional hazards model, adjusting for the above-mentioned factors. *P* < 0.05 was considered statistically significant for all analyses, and all *P* values were two-tailed.

## Results

### Clinical characteristics and follow-up

A total of 870 eligible non-M3 AML patients including 476 men (54.71%) and 394 women were enrolled in this study. Clinical characteristics of the patients were summarized in Table 1. The median age was 43 years (range 14–79 years) for the patients, and 103 patients (11.84%) aged 60 ≥ years. According to the FAB subtype criteria for AML, most patients were classified as M2 subtype (51.15%), followed by M4 (20.69%) and M5 (19.89%). Data for risk stratification was available for 759 patients: 202 patients with a favorable-risk, 384 with an intermediate-risk, and 173 with a poor-risk. A total of 479 AML patients (60.71%, 479/789) showed normal karyotype, and 96 patients (13.10%, 96/733) carrying *FLT3*-*ITD* mutations.

**Table 1:** Clinical features of AML patients according to *DNMT3A* R882 mutation status

Clinical features	Total (n = 870)	R882 wild-type (n = 796)	R882 mutation (n = 74)	*P* value
Age^a^, years	42 ± 15	41 ± 15	48 ± 11	*6.64 × 10* ^*−6*^
Age ≥ 60 years, n (%)	103 (11.84)	94 (11.81)	9 (12.16)	0.928
Male, n (%)	476 (54.71)	443 (55.65)	33 (44.59)	0.068
FAB classification, n (%)				
M2	445 (51.15)	428 (53.77)	17 (22.97)	*3.99 × 10* ^*−7*^
M4	180 (20.69)	162 (20.35)	18 (24.32)	0.420
M5	173 (19.89)	139 (17.46)	34 (45.95)	*4.31 × 10* ^*−9*^
Other subtypes or undetermined	72 (8.28)	67 (8.42)	5 (6.76)	
Parameters at diagnosis^a^				
WBC count, ×10^9^/L	39.19 ± 62.25	37.56 ± 62.48	56.72 ± 57.27	*0.012*
RBC count, ×10^12^/L	2.32 ± 1.53	2.32 ± 1.58	2.25 ± 0.66	0.710
Hemoglobin, g/L	73.48 ± 21.11	73.45 ± 21.50	73.82 ± 16.36	0.858
Platelets count, ×10^9^/L	58.21 ± 83.94	55.99 ± 84.15	82.71 ± 78.02	*0.010*
Neutrophil count, ×10^9^/L	12.69 ± 32.04	12.53 ± 32.67	14.45 ± 24.10	0.630
LDH, U/L	555.17 ± 694.61	553.00 ± 713.95	578.29 ± 441.34	0.771
Bone marrow blasts, %	64.23 ± 21.40	63.97 ± 21.50	67.08 ± 20.19	0.244
Risk stratifications^b^, n (%)	n = 759^c^	n = 696	n = 63	
Intermediate risk	384 (50.59)	355 (51.01)	29 (46.03)	
Low risk	202 (26.61)	190 (27.30)	12 (19.05)	0.467^d^
High risk	173 (22.79)	151(21.70)	22 (34.92)	0.051^d^
FLT3-ITD, n (%)	n = 733^c^	n = 670	N = 63	
Negative	637 (86.90)	591 (88.21)	46 (73.02)	
Positive	96 (13.10)	79 (11.79)	17 (26.98)	*0.001*
Karyotype, n (%)	n = 789^c^	n = 721	n = 68	
Normal cytogenetics	479 (60.71)	425 (58.95)	54 (79.41)	
Non-normal cytogenetics	310 (39.29)	296 (41.05)	14 (20.59)	*0.001*
HSCT, n (%)	161 (18.51)	151 (18.97)	10 (13.51)	0.248

*FAB classification* French–Britain–American classification, *WBC* white blood cell, *RBC* red blood cell, *LDH* lactate dehydrogenase, *HSCT* hematopoietic stem cell transplantation

Italic values indicate significance of *P* value (*P* < 0.05)

^a^Data are presented as mean ± standard deviation (SD) for continuous variable

^b^Risk stratification based on NCCN guidelines version 1.2015 acute myeloid leukemia

^c^Number of patients was based on the available clinical information

^d^Intermediate risk was served as a reference

The CR rate after the first cycle of induction therapy was 40.32% (357/864), and 602 (69.68%, 602/864) patients achieved CR after 2 cycles of induction chemotherapy. Efficacy for induction therapy was not assessed for 6 patients (Table 2). One hundred and sixty-one patients (18.51%) received HSCT after achievement of CR, and 414 (47.59%) patients died by the end of the follow-up period. With a median follow-up of 315 days (range 25–2500 days), the median OS was 607 days. For the 688 patients achieved CR ultimately after one or more cycles of induction therapy, the median DFS was 491 days, and 256 (37.21%, 256/688) patients relapsed during the follow-up period.

**Table 2:** Comparison of CR rates between *DNMT3A* R882 mutation and wild type patients after one or two courses of induction therapy

Chemotherapy cycles	Total CR/n (%)	R882 wild-type CR/n (%)	R882 mutation CR/n (%)	OR (95% CI)	*P* value
One cycle	357/864 (41.32)	336/791 (42.48)	21/73 (28.77)	1.829 (1.081–3.094)	*0.023*
Two cycles	601/864 (69.56)	556/791 (70.29)	45/73 (61.64)	1.472 (0.897–2.417)	0.124

*CR* complete response, *R882* arginine 882, *OR* odds ratio, *CI* confidence interval

Italic values indicate significance of *P* value (*P* < 0.05)

### Comparison of clinical features between *DNMT3A* R882 mutation groups

As shown in Table 1 and Additional file 1: Table S2, 74 AML patients (8.51%) carried the *DNMT3A* R882 mutations, among which 54 cases were positive for the R882H mutation, 18 cases with the R882C mutation, and 2 cases with the R882P (*DNMT3A* c.2645G > C) mutation. The distribution of R882 mutant-allele ratio was skewed to the left, and the median was 38.5% (Additional file 2: Figure S1). Patients with the *DNMT3A* R882 mutant were significantly older (*P *= 6.64 × 10^−6^), showed significantly higher WBC (*P *= 0.012) and platelet counts (*P *= 0.010) at disease diagnosis. No significant difference in gender, antecedent hematologic disorder, RBC (red blood cells) and neutrophil counts, hemoglobin and lactate dehydrogenase (LDH) levels, or percentages of blast cells in bone marrow was observed at diagnosis between patients with and without the *DNMT3A* R882 mutations. Patients positive for the *DNMT3A* R882 mutations were less frequent in the M2 (22.97% vs. 53.77%, *P* = 3.99 × 10^−7^) but over-represented in the M5 subtype (45.95% vs. 17.46%, *P* = 4.31 × 10^−9^). *FLT3*-*ITD* mutations (*P *= 0.001) and normal karyotype (*P *= 0.001) occurred more frequently in patients positive for the *DNMT3A* R882 mutations. No difference in the proportion of risk stratification status based on cytogenetics and molecular abnormalities was observed between the *DNMT3A* R882 variants positive and negative patients.

### Comparison of response to induction chemotherapy according to *DNMT3A* R882 mutant status

Association between *DNMT3A* R882 mutations and chemosensitivity after one or two induction were analyzed. Compared to patients without R882 mutations, those *DNMT3A* R882 mutations positive patients showed a significantly lower CR rate after the first cycle of induction therapy (28.77% vs. 42.48%, *P* = 0.023), but the difference disappeared after two cycles of induction (61.64% vs. 70.29%, *P* = 0.124, Table 2). Results of multivariate analysis showed that age at diagnosis and AML risk stratification were associated with non-CR risk significantly after two-courses of induction, and risk stratification status was also associated with response to one cycle of induction significantly. However, there was no association between *DNMT3A* R882 mutations and non-CR risk after one and two cycles of induction chemotherapy when adjusted by AML prognostic factors (Additional file 1: Table S3). No significant difference in incidence of TRM between patients with and without *DNMT3A* R882 mutations was observed as well (Additional file 1: Table S4).

We then analyzed the impact of types of R882 mutation and allelic ratio of R882 mutations on CR rates. As shown in Additional file 1: Table S5, no significant difference in CR rates after one and two cycles of induction therapy was observed between R882H and R882C mutations. When allele ratio of *DNMT3A* R882 mutants was considered, no significant difference in CR rates after one and two cycles of induction between high and low allele ratio groups was found. Yet, significantly lower CR rates after one and two courses of induction therapy were observed in patients with high mutation allele ratio as compared to those R882 mutation negative patients (24.32% vs. 42.48%, *P* = 0.029 for the first cycle of induction therapy; 54.05% vs. 70.29%, *P* = 0.036 for the second cycle of induction therapy, respectively, Additional file 1: Table S5).

### Influence of *DNMT3A* R882 mutations on chemosensitivity to anthracyclines

Given that the negative impact of *DNMT3A* mutations could be dependent on both the dosage and types of anthracyclines [26, 27], stratification analysis based on specific drugs of the anthracyclines in induction chemotherapy was further carried out. In the subgroup of patients receiving aclarubicin in the first cycle of induction therapy (n = 187), carriers of *DNMT3A* R882 mutations had a significantly lower CR rate than R882 mutant negative cases (21.43% vs. 53.18%, *P* = 0.022). Similarly, in the subgroup of patients who received aclarubicin in either the first or the second cycles of induction therapy (n = 379), a significantly decreased CR rate after two-courses of induction therapy was observed in R882 mutant positive cases (n = 30) as compared with the mutation negative group (46.67% vs. 65.62%, *P* = 0.038, Table 3). Moreover, patients with *DNMT3A* R882 mutations showed inferior prognosis in patients receiving aclarubicin in either the first or the second cycles of induction therapy (Additional file 3: Figure S2).

**Table 3:** CR rates between *DNMT3A* R882 mutation and wild type patients after one or two cycles of induction based on different regimen

Induction cycles	Anthracyclines	Total CR/n (%)	R882 wild-type CR/n (%)	R882 mutation CR/n (%)	OR (95% CI)	*P* value
First cycle	Aclarubicin	95/187 (50.80)	92/173 (53.18)	3/14 (21.43)	4.165 (1.122–15.451)	*0.022*
	Daunorubicin	28/84 (33.33)	27/78 (34.62)	1/6 (16.67)	2.647 (0.294–23.821)	0.653
	Idarubicin	90/203 (44.33)	83/183 (45.35)	7/20 (35.00)	1.541 (0.589–4.041)	0.376
	Mitoxantrone	114/287 (39.72)	107/264 (40.53)	7/23 (30.43)	1.558 (0.620–3.915)	0.343
	Pirarubicin	21/74 (28.38)	20/69 (28.99)	1/5 (20.00)	1.633 (0.172–15.524)	1.000
First or second cycle	Aclarubicin	243/379 (64.12)	229/349 (65.62)	14/30 (46.67)	2.181 (1.030–4.619)	*0.038*

*CR* complete response, *R882* arginine 882, *OR* odds ratio, *CI* confidence interval

Italic values indicate significance of *P* value (*P* < 0.05)

### Influence of *DNMT3A* R882 mutations on AML prognosis

**Fig. 1:**
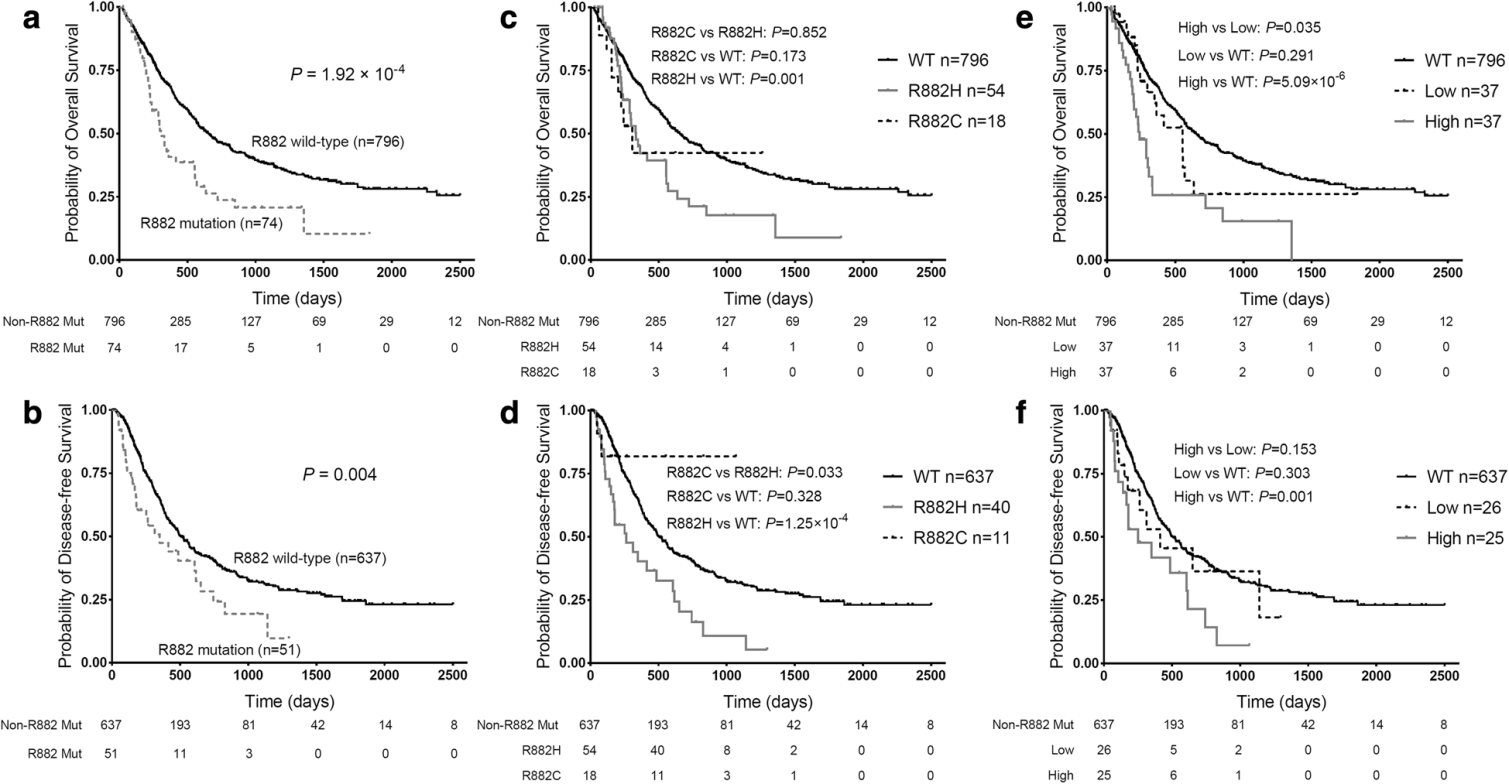
Comparison of overall survival (OS) and disease-free survival (DFS) according to *DNMT3A* R882 status in AML patients. **a**, **b** Impact of *DNMT3A* R882 mutations on OS (**a**) and DFS (**b**) in AML patients. **c**, **d** Impact of *DNMT3A* R882 mutant types on OS (**c**) and DFS (**d**) in AML patients. **e**, **f** Impact of allele ratio of *DNMT3A* R882 mutation on OS (**e**) and DFS (**f**) in AML patients

Our previous studies have reported shorter OS in *DNMT3A* R882 mutant AML patients [19, 20]. In this study, we also observed that patients with the R882 mutations had significantly shorter OS and DFS than R882 mutant negative patients (*P* = 1.92 × 10^−4^; *P* = 0.004, respectively). In detail, the median OS and DFS respectively were 305 days (range 248–362 day) and 350 days (range 157–543 day) for carriers of *DNMT3A* R882 mutations, but were 656 days (range 556–756 day) and 508 days (range 430–586 day) for R882 mutant negative cases (Fig. 1a, b). Analysis with proportional hazards model indicated that *DNMT3A* R882 mutations were significantly and independently associated with inferior OS and DFS in AML patients (HR = 1.725, 95% CI 1.221–2.437, *P *= 0.002 for OS; HR = 1.694, 95% CI 1.114–2.577, *P *= 0.014 for DFS, Table 4). Besides, the results showed that older age, increased WBC count and high-risk stratification were associated with poorer OS, while low-risk stratification was related to better OS in AML patients. The association of AML risk stratifications and WBC count with DFS was also observed in the Cox model (Table 4). When the patients were stratified based on *DNMT3A* R882 mutation types, patients with the R882H mutation also showed significantly worse OS (*P* = 0.001) and DFS (*P* = 1.25 × 10^−4^) as compared with the R882 mutation negative group (Fig. 1c, d).

**Table 4:** *DNMT3A* R882 mutations and clinical factors in Cox regression analysis for AML prognosis

Variables in the model	Overall survival (n = 870)		Disease-free survival (n = 688)	
	HR (95% CI)	*P* value	HR (95% CI)	*P* value
*DNMT3A* R882 mutations	1.725 (1.221–2.437)	*0.002*	1.694 (1.114–2.577)	*0.014*
Age, years	1.018 (1.010–1.026)	*7.47 × 10* ^*−6*^	1.005 (0.997–1.014)	0.229
WBC count, ×10^9^/L	1.002 (1.001–1.004)	*0.007*	1.002 (1.000–1.004)	0.051
Platelets count, ×10^9^/L	0.997 (0.995–0.999)	*0.011*	0.999 (0.997–1.001)	0.159
Risk stratifications		*6.80 × 10* ^*−22*^		*9.13 × 10* ^*−7*^
Low vs. intermediate	0.681 (0.512–0.907)	*0.009*	0.693 (0.522–0.920)	*0.011*
High vs. intermediate	2.729 (2.146–3.469)	*2.53 × 10* ^*−16*^	1.794 (1.323–2.433)	*1.68 × 10* ^*−4*^

*HR* hazard ratios, *CI* confidence interval, *R882* arginine 882, *WBC* white blood cell

Italic values indicate significance of *P* value (*P* < 0.05)

Give that mutations of *DNMT3A* R882, *FLT3*-ITD and *NPM1* cooperated with each other in leukemogenesis and chemotherapy resistance [35], we further compared the prognosis between patients with single and double somatic mutations base on the available data (Additional file 4: Figure S3). In comparison with cases with *FLT3*-ITD but no R882 mutations, patients carrying both *FLT3*-ITD and R882 mutations showed significantly inferior OS (*P* = 0.041), but carriers of R882 mutations alone showed marginally better OS (*P* = 0.089, Additional file 4: Figure S3A). Similarly, AML patients with *FLT3*-ITD but no mutation in the 12th exon of *NPM1* showed significantly favorable OS than those carrying the two mutations simultaneously (*P* = 0.010), but showed significantly shorter OS and DFS than carriers of the *NPM1* mutations alone (*P* = 0.002; *P* = 0.001, respectively, Additional file 4: Figure S3E, F).

### Effect of *DNMT3A* R882 mutation allele ratio on prognosis of AML

Next, we investigated the influence of the allele ratio of *DNMT3A* R882 mutation on prognosis of AML. *DNMT3A* R882 mutant positive patients were divided into two groups based on the median of R882 mutant allele ratio. Compared with patients with lower R882 mutant allele ratio (≤ 0.39, n = 37), the median OS of the patients with a higher R882 mutant allele ratio (> 0.39, n = 37) was significantly shorter (237 vs. 553 days, *P* = 0.035, Fig. 1e). Similarly, the median DFS was marginally shorter in patients with a higher R882 mutant allele ratio compared with those with a lower R882 mutant allele ratio (252 vs. 413 days, *P* = 0.153, Fig. 1f). After adjusting for AML prognosis factors, *DNMT3A* R882 mutant allele ratio was marginally associated with poorer OS and DFS (HR = 1.029, 95% CI 0.998–1.061, *P* = 0.066 for OS; HR = 1.026, 95% CI 0.990–1.063, *P* = 0.159 for DFS, Table 5). In addition, patients with high mutant allele ratio showed a significantly shorter OS and RFS than those without the *DNMT3A* R882 mutations (*P* = 5.09 × 10^−6^, *P* = 0.001), while no difference in disease prognosis was observed between the low allele ratio and the R882 mutant negative patients (Fig. 1e, f).

**Table 5:** Allelic ratio of *DNMT3A* R882 mutations and clinical factors in Cox regression analysis for OS and DFS

Variables in the model	Overall survival (n = 74)		Disease-free survival (n = 51)	
	HR (95% CI)	*P* value	HR (95% CI)	*P* value
*DNMT3A* R882 mutation ratio, %	1.029 (0.998–1.061)	0.066	1.026 (0.990–1.063)	0.159
Age, years	1.047 (1.006–1.090)	*0.025*	1.031 (0.984–1.081)	0.197
WBC count, ×10^9^/L	1.005 (0.999–1.010)	0.088	1.005 (0.998–1.012)	0.171
Platelets count, ×10^9^/L	0.999 (0.995–1.003)	0.757	1.001(0.996–1.005)	0.709
Risk stratification		*0.027*		*0.032*
Low vs. intermediate	1.055 (0.362–3.075)	0. 921	0.618 (0.185–2.071)	0.436
High vs. intermediate	2.758 (1.288–5.904)	*0.009*	3.351 (1.183–9.494)	*0.023*

*HR* hazard ratios, *CI* confidence interval, *R882* arginine 882, *WBC* white blood cell

Italic values indicate significance of *P* value (*P* < 0.05)

## Discussion

In this cohort study, we observed the influence of *DNMT3A* R882 mutations and the allele ratio of the mutations on disease prognosis in Chinese AML patients. We confirmed *DNMT3A* R882 mutations as independent predictors of AML poor prognosis, especially in patients with higher R882 mutant allele ratio. In addition, we observed that carriers of the *DNMT3A* R882 mutations showed higher WBC and platelet counts at diagnosis, and were over-represented in elder patients or those with the *FLT3*-*ITD* somatic mutation. Further, we found that the *DNMT3A* R882 mutations were associated with chemoresistance to “7 + 3” induction chemotherapy consisted of aclarubicin.

We replicated the previous reports about the influence of *DNMT3A* R882 mutations on AML prognosis in this study [10, 17–19]. The disease prognosis of AML has not been substantially improved in recent decades [2], and the major challenge for AML treatment management is the disease heterogeneity. Although the prognostic significance of some cytogenetics and molecular abnormalities have been confirmed, it is far from enough for AML prognosis management. We suggested that the *DNMT3A* R882 mutations could be considered for AML risk stratification system and treatment decision.

The highlight of our study is that we investigated the impact of the allele ratio of R882 mutations on response to induction chemotherapy and disease prognosis of AML patients for the first time. We observed that AML patients with higher R882 mutant allele ratio showed significantly worse response to induction therapy and prognosis compared with the R882 mutation negative patients, whereas no difference in CR rates, DFS or OS between patients with lower R882 mutant allele ratio and wild type patients was observed. Moreover, patients with higher R882 mutant allele ratio also showed significantly decreased OS compared with those with lower mutant allele ratio, and allele ratio of the mutation was an independent prognostic factor of AML in the Cox model. The association between higher allele ratios of *FLT3*-*ITD* or *NPM1* somatic mutations and an inferior prognosis of AML has been reported [28, 30], which could cooperate with *DNMT3A* mutations for leukemogenesis and drug-resistance [35]. The clinical significances of *DNMT3A* mutation allele ratio might be explained by cancer biology. The mutations with lower allele might derive from a minor subclone, which means occurrence of the mutations at later-stage in the leukemogenic process. Loss of DNMT3A in HSCs increases the ability of self-renewal, inhibits differentiation [14]. And, the stem cells and minor subclone with *DNMT3A* mutations, unlikely to be detected in bone narrow of AML patients, may be critically important to clinical outcomes. Therefore, single cell analysis is suggested to evaluate differential behaviors of sub-clones carrying the mutations at different stages of AML in future research [36]. These results implicated that the presence or absence of *DNMT3A* R882 mutations did not integrally and effectively predict clinical outcomes of AML, and its mutant allele burden also should be taken into account in order to obtain a more precise and comprehensive assessment of the prognostic risk stratification for AML.

In respect of *DNMT3A* R882 mutant types, we found AML patients with R882H mutation had a shorter OS and DFS relative to R882 wildtype patients. However, no significant difference in a majority of clinical outcomes between R882C mutant group and R882H or R882 wildtype groups was observed, which might arise from small sample size of the R882C group. In general, we thought that clinical significance of the R882C mutation might be similar to the R882H in AML. Of course, further studies with larger sample size are needed to investigate the impact of R882C mutation on AML, and the specific molecular mechanisms of R882C in AML is also worth exploring.

More importantly, we carried out a subgroup analysis based on induction regimens and noted that carriers of *DNMT3A* R882 mutations had an inferior response to aclarubicin combined therapy compared with R882 wildtype AML patients. Studies have shown that anthracyclines rather than to etoposide, both of which are topoisomerase II inhibitors, could evict histone from open chromatin in blast cells from AML patients resulting in cytotoxicity [37]. Besides, aclarubicin intercalation facilitates nucleosome turnover around promoters by its effect on DNA topology thereby killing cancer cells [38]. But aclarubicin does not bring about DNA double strand breaks when inhibiting topoisomerase II [39]. Those studies indicated that aclarubicin enhances histone eviction, whereas etoposide inhibits topoisomerase II, but daunorubicin exerts its cytotoxic effect by both above-mentioned mechanisms [40]. Recently, Guryanova et al. observed reduced sensitivity of AML cell lines with R882 mutations to anthracyclines, especially aclarubicin, through attenuated nucleosome eviction in response to cytotoxic chemotherapy, but not to etoposide [35]. Therefore, *DNMT3A* R882 mutations might drive a stronger chemoresistance to aclarubicin than other anthracycline drugs, which are consistent with the results of our study. Aclarubicin is mostly used in CAG regimen, which consists of cytarabine, aclarubicin, and granulocyte colony-stimulating factor (G-CSF). The regimen is effective and has been widely applied to treat AML in China, particularly for high-risk patients [41]. The CR rate of patients receiving regiments including aclarubicin in the first cycle of induction also reached 50.8% in our cohort, which was higher than other anthracyclines. In clinical practice, the adverse reaction of myelosuppressive was considered to be severe for aclarubicin, and G-CSF is usually used concomitantly to reduce or avoid myelosuppression. We suggest further prospective trials or studies based on our findings to replicate these findings and to evaluate the use of aclarubicin for AML patients with *DNMT3A* R882 mutations.

There is no consensus of opinion among studies as to the impact of *DNMT3A* R882 mutations on prognosis and chemosensitivity of AML [19, 42]. Given that no difference in AML clinical outcomes between lower mutation allele and R882 wild type patients was observed in the study, the inconsistency probably stems from lack of consideration of allele ratio of *DNMT3A* R882 mutations in those studies. In addition, ignoring therapy regimens of AML patients might interfere with the comparability of cohorts. Therefore, subgroup analyses based on induction therapy regimens are imperative for comparison of response to induction chemotherapy between different R882 status groups. Because AML is a highly heterogeneous disease affected by pathogenesis, clinical indicators and genetic polymorphisms [31, 43–46], explaining the AML prognosis and drug response would likely require a more comprehensive approach.

## Conclusion

In summary, *DNMT3A* R882 mutations were associated with a worse prognosis in Chinese AML patients, but the influence depended on *DNMT3A* R882 mutant-allele ratio and patients with higher allele ratio had a worse prognosis. These findings suggest that *DNMT3A* R882 mutations and its mutant alleles may be useful for risk stratification in AML patients. In addition, the association of *DNMT3A* R882 mutations with an inferior response to induction therapy with aclarubicin was found in Chinese AML patients, which could provide new insightful information for AML individualized chemotherapy.

## Additional files

**Additional file 1: Table S1.** Primer sequences used for detection of somatic mutation in AML. **Table S2.** Clinical features of AML patients according to *DNMT3A* R882 mutation status (supplementary). **Table S3.** *DNMT3A* R882 mutations and clinical factors of logistic regression analysis for chemosensitivity of AML. **Table S4.** Treatment-related mortalities of AML patients after one or two cycles induction therapy based on *DNMT3A* R882 status. **Table S5.** Effects of *DNMT3A* R882 mutation type and allelic ratio on AML CR rates after one or two cycles of induction therapy.

**Additional file 2: Figure S1.** Histogram of distribution of *DNMT3A* R882 mutant-allele ratio in AML patients.

**Additional file 3: Figure S2.** Impact of *DNMT3A* R882 mutations on overall survival (OS) and disease-free survival (DFS) in the AML patients treated with aclarubicin. (A, B) Comparison of OS (A) and DFS (B) between R882 mutations and wild type groups in the AML patients receiving aclarubicin in the first cycle of induction therapy. (C, D) Comparison of OS (C) and DFS (D) based on R882 status in the AML patients receiving aclarubicin in the first or second cycle of induction.

**Additional file 4: Figure S3.** Interaction effect of *DNMT3A* R882, *FLT3*-ITD and *NPM1* mutations on overall survival (OS) and disease-free survival (DFS) in AML patients. (A, B) Comparison of OS (A) and DFS (B) among patients with one or both of R882 and *FLT3*-ITD mutations. (C, D) Comparison of OS (C) and DFS (D) among patients with one or both of R882 and *NPM1* mutations. (E, F) Comparison of OS (E) and DFS (F) among patients with one or both of *FLT3*-ITD and *NPM1* mutations.

## Data Availability

The datasets supporting the conclusions of this article are included within the article and its additional files.

## Abbreviations

*DNMT3A*: DNA methyltransferase 3 alpha
R882: arginine 882
AML: acute myeloid leukemia
OS: overall survival
DFS: disease-free survival
*NPM1*: Nucleophosmin 1
*CEBPA*: CCAAT/enhancer binding protein alpha
*c*-*KIT*: tyrosine-protein kinase Kit
*FLT3*: Fms-like tyrosine kinase 3
FAB: French–Britain–American
CR: complete remission
*FLT3*-*ITD*: FLT3 internal tandem duplication
ITD: internal tandem duplication
*RUNX1*: runt related transcription factor 1
T-AML: therapy-related AML
TRM: treatment-related mortality
HSCT: hematopoietic stem cell transplantation
PCR: polymerase chain reaction
OR: odds ratio
HR: hazard ratios
WBC: white blood cell
RBC: red blood cells
LDH: lactate dehydrogenase
