# Supplementary material for: Influence of DNMT3A R882 mutations on AML prognosis determined by the allele ratio in Chinese patients
Source: J Transl Med. 2019 Jul 10;17:220. doi: 10.1186/s12967-019-1959-3 (PMC6621981; doi:10.1186/s12967-019-1959-3)
Supplement: Supplementary file 1 — Additional file 1: Table S1. Primer sequences used for detection of somatic mutation in AML. Table S2. Clinical features of AML patients according to DNMT3A R882 mutation status (supplementary). Table S3. DNMT3A R882 mutations and clinical factors of logistic regression analysis for chemosensitivity of AML. Table S4. Treatment-related mortalities of AML patients after one or two cycles induction therapy based on DNMT3A R882 status. Table S5. Effects of DNMT3A R882 mutation type and allelic ratio on AML CR rates after one or two cycles of induction therapy. [file 12967_2019_1959_MOESM1_ESM.docx]

**Table S1.** Primer sequences used for detection of somatic mutation in AML

| **Mutations** | **Forward Primer (5’-3’)** | **Reverse Primer (5’-3’)** | **Pyrosequencing Primer (5’-3’)** |
| --- | --- | --- | --- |
| *FLT-ITD* | GCAATTTAGGTATGAAAGCCAGC | CTTTCAGCATTTTGACGGCAACC |  |
| *NPM1* | Biotin-TTTTTTTCCAGGCTATTCAAGATC | GGGAAAGTTCTCACTCTGCAT | TTAAAGAGACTTCCTCCAC |
| R882 *DNMT3A* | Biotin-GGTCCTGCTGTGTGGTTAGACG | AAGAGGTGGCGGATGACTG | CTCTGCCTCGCCAAG |

**Table S2.** Clinical features of AML patients according to *DNMT3A* R882 mutation status (supplementary).

| **Clinical Features** | **Total**  **(n=870)** | **R882 Wild-type**  **(n = 796)** | **R882 Mutation**  **(n = 74)** | ***P* Value** |
| --- | --- | --- | --- | --- |
| AML type, n (%) |  |  |  |  |
| *De novo* AML | 826 (94.94) | 754 (94.72) | 72 (97.30) | 0.491^†^ |
| AML after prior MDS | 44 (5.06) | 42 (5.28) | 2 (2.70) |  |
| Karyotype, n (%) |  |  |  |  |
| inv(16), t(16;16) | 26 (2.99) | 26 (3.27) | - | 0.222 |
| t(8;21) | 100(11.49) | 100 (12.56) | - | **0.001** |
| Normal cytogenetics | 479 (55.06) | 425 (53.39) | 54 (72.97) | **0.001** |
| +8 alone | 14 (1.61) | 13 (1.63) | 1 (1.35) | 1.000 |
| t(9;11) | 6 (0.69) | 6 (0.75) | - | 1.000 |
| Other non-defined | 71 (8.16) | 64 (8.04) | 7 (9.46) | 0.670 |
| Complex (3 or more abnormalities) | 54 (6.21) | 50 (6.28) | 4 (5.41) | 0.963 |
| -5, 5q-, -7, 7q- | 16 (1.84) | 15 (1.88) | 1 (1.35) | 1.000 |
| 11q23-non t(9;11) | 13 (1.49) | 13 (1.63) | - | 0.544 |
| inv(3), t(3;3) | 5 (0.57) | 4 (0.50) | 1 (1.35) | 0.360 |
| t(6;9) | 6 (0.69) | 6 (0.75) | - | 1.000 |
| t(9;22) | 2 (0.23) | 2 (0.25) | - | 1.000 |
| Undetermined | 81 (9.31) | 75 (9.42) | 6 (8.11) |  |
| Anthracyclines in first induction, n (%) |  |  |  |  |
| Aclarubicin | 191 (21.95) | 176 (22.11) | 15 (20.27) | 0.715 |
| Daunorubicin | 84 (9.66) | 78 (9.80) | 6 (8.11) | 0.638 |
| Idarubicin | 204 (23.45) | 184 (23.12) | 20 (27.03) | 0.447 |
| Mitoxantrone | 288 (33.10) | 265 (33.29) | 23 (31.08) | 0.699 |
| Pirarubicin | 74 (8.50) | 69 (8.67) | 5 (6.76) | 0.573 |
| Other regimens | 29 (3.33) | 24 (3.02) | 5 (6.76) |  |

**Table S3.** *DNMT3A* R882 mutations and clinical factors of logistic regression analysis for chemosensitivity of AML.

| **Variables in the Model** | **Non-CR Risk after One Cycle of Induction** | |  | **Non-CR Risk after Two Cycle of Induction** | |
| --- | --- | --- | --- | --- | --- |
|  | **OR (95%CI)** | ***P* Value** |  | **OR (95%CI)** | ***P* Value** |
| R882 *DNMT3A* mutations | 1.330 (0.744-2.377) | 0.336 |  | 0.961 (0.533-1.731) | 0.894 |
| Age, years | 1.007 (0.996-1.018) | 0.209 |  | 1.016 (1.004-1.028) | **0.009** |
| WBC count, ×10^9^/L | 1.002 (0.999-1.005) | 0.121 |  | 1.003 (1.000-1.005) | 0.059 |
| Risk stratification |  | **1.35 × 10^-4^** |  |  | **4.64 × 10^-11^** |
| Low *vs* intermediate | 0.607 (0.428-0.860) | **0.005** |  | 0.442 (0.280-0.696) | **4.35 × 10^-4^** |
| High *vs* intermediate | 1.542 (1.038-2.289) | **0.032** |  | 2.477 (1.692-3.624) | **3.03 × 10^-6^** |

Abbreviation: CR, complete response; OR, odds ratio; CI, confidence interval; R882, arginine 882.

**Table S4.** Treatment-related mortalities of AML patients after one or two cycles induction therapy based on *DNMT3A* R882 status.

| **Chemotherapy Cycles** | **Total**  **TRM/n (%)** | **R882 Wild-type**  **TRM/n (%)** | **R882 Mutation**  **TRM/n (%)** | **OR (95%CI)** | ***P* Value** |
| --- | --- | --- | --- | --- | --- |
| One cycle | 32/870 (3.68) | 28/796 (3.52) | 4/74 (5.41) | 1.567 (0.534-4.596) | 0.615 |
| Two cycles | 47/870 (5.40) | 42/796 (5.28) | 5/74 (6.76) | 1.472 (0.897-2.417) | 0.787 |

Abbreviation: TRM, treatment-related mortality; R882, arginine 882; OR, odds ratio; CI, confidence interval.

**Table S5.** Effects of *DNMT3A* R882 mutation type and allelic ratio on AML CR rates after one or two cycles of induction therapy.

| **Chemotherapy Cycles** | **R882 *DNMT3A* Status** | **CR/n, (%)** | **R882 Mutation Subtype**  ***vs* R882 Wild-type** | |  | **Intercomparison between**  **R882 Subtype Mutations** | |
| --- | --- | --- | --- | --- | --- | --- | --- |
|  |  |  | **OR (95%CI)** | ***P* value** |  | **OR (95%CI)** | ***P* value** |
| One cycle | R882 wild-type | 336/791 (42.48) | 1.000 (reference) |  |  |  |  |
|  | R882H mutation | 18/53 (33.96) | 1.436 (0.799-2.579) | 0.224 |  | 1.000 (reference) |  |
|  | R882C mutation | 3/18 (16.67) | 3.692 (1.060-12.856) | **0.028** |  | 2.571 (0.658-10.056) | 0.165 |
|  | Low R882 mutation ratio | 12/36 (33.33) | 1.477 (0.728-2.995) | 0.277 |  | 1.000 (reference) |  |
|  | High R882 mutation ratio | 9/37 (24.32) | 2.297 (1.070-4.933) | **0.029** |  | 1.556 (0.560-4.322) | 0.395 |
| Two cycles | R882 wild-type | 556/791 (70.29) | 1.000 (reference) |  |  |  |  |
|  | R882H mutation | 35/53 (66.04) | 1.217(0.675-2.192) | 0.513 |  | 1.000 (reference) |  |
|  | R882C mutation | 10/18 (55.56) | 1.893 (0.738-4.856) | 0.178 |  | 1.556 (0.523-4.625) | 0.425 |
|  | Low R882 mutation ratio | 25/36 (69.44) | 1.041(0.504-2.150) | 0.913 |  | 1.000 (reference) |  |
|  | High R882 mutation ratio | 20/37(54.05) | 2.011 (1.035-3.908) | **0.036** |  | 1.932(0.740-5.044) | 0.176 |

Abbreviation: CR, complete response; OR, odds ratio; CI, confidence interval; R882, arginine 882; R882H, *DNMT3A* c.2645G>A; R882C *DNMT3A* c.2644C>T.
