# Supplementary figures and images for: Influence of DNMT3A R882 mutations on AML prognosis determined by the allele ratio in Chinese patients
Source: J Transl Med. 2019 Jul 10;17:220. doi: 10.1186/s12967-019-1959-3 (PMC6621981; doi:10.1186/s12967-019-1959-3)

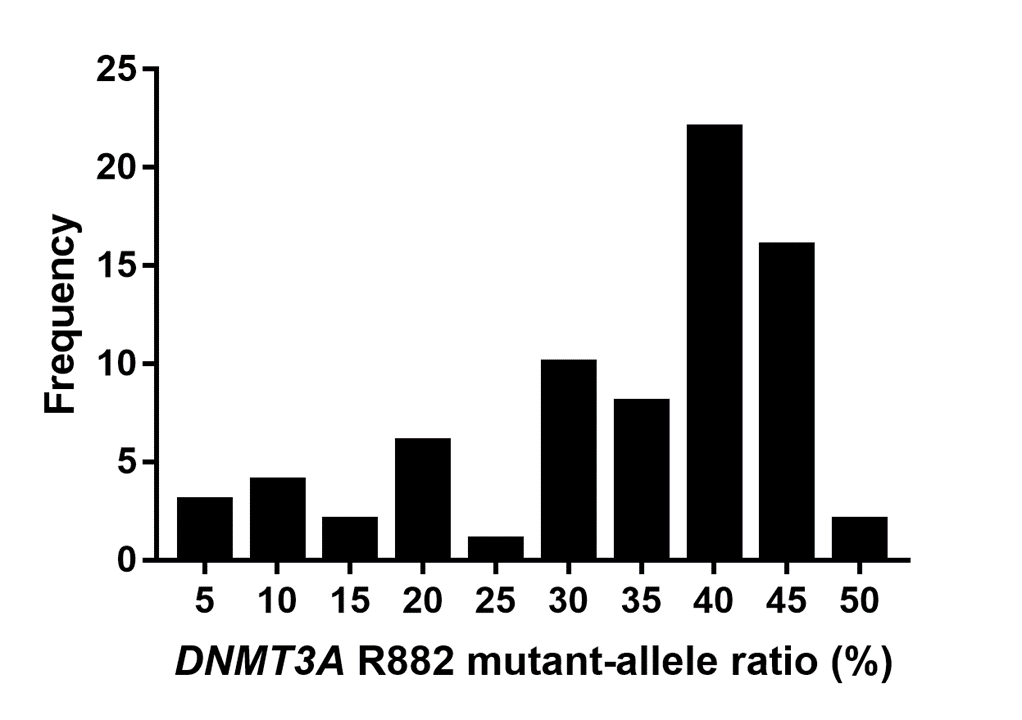

Supplement: Supplementary file 2 — Additional file 2: Figure S1. Histogram of distribution of DNMT3A R882 mutant-allele ratio in AML patients. [file 12967_2019_1959_MOESM2_ESM.png]

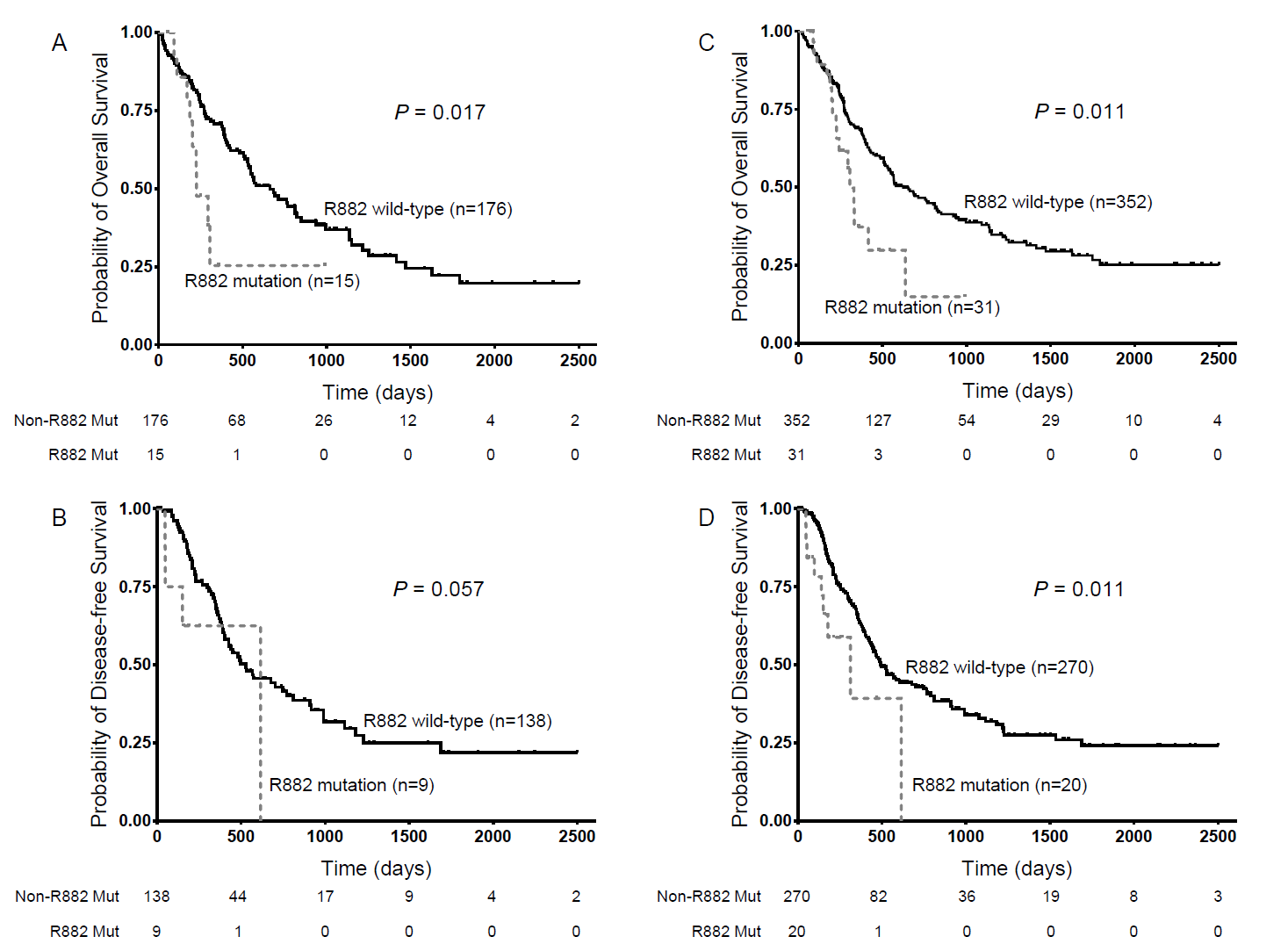

Supplement: Supplementary file 3 — Additional file 3: Figure S2. Impact of DNMT3A R882 mutations on overall survival (OS) and disease-free survival (DFS) in the AML patients treated with aclarubicin. (A, B) Comparison of OS (A) and DFS (B) between R882 mutations and wild type groups in the AML patients receiving aclarubicin in the first cycle of induction therapy. (C, D) Comparison of OS (C) and DFS (D) based on R882 status in the AML patients receiving aclarubicin in the first or second cycle of induction. [file 12967_2019_1959_MOESM3_ESM.png]

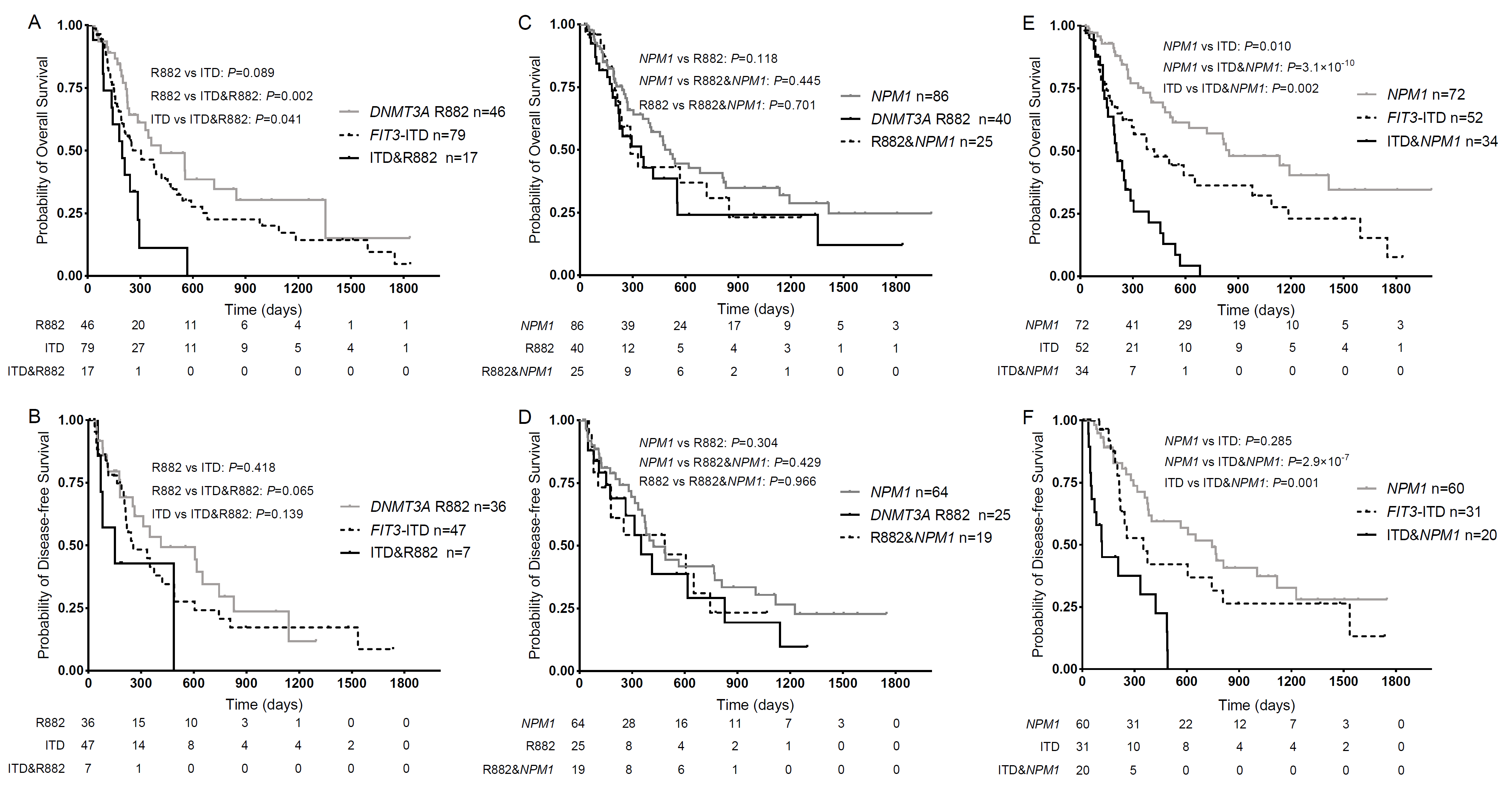

Supplement: Supplementary file 4 — Additional file 4: Figure S3. Interaction effect of DNMT3A R882, FLT3-ITD and NPM1 mutations on overall survival (OS) and disease-free survival (DFS) in AML patients. (A, B) Comparison of OS (A) and DFS (B) among patients with one or both of R882 and FLT3-ITD mutations. (C, D) Comparison of OS (C) and DFS (D) among patients with one or both of R882 and NPM1 mutations. (E, F) Comparison of OS (E) and DFS (F) among patients with one or both of FLT3-ITD and NPM1 mutations. [file 12967_2019_1959_MOESM4_ESM.png]
